# A community-focused rural academic health department model in North Carolina: elevating community engagement, rural public health practice and research

**DOI:** 10.3389/fpubh.2026.1893009

**Published:** 2026-09-03

**Authors:** Carmen D. Samuel-Hodge, Lisa Macon Harrison, Wendy Smith, Karl T. Johnson, Anna Feng, Devon Noonan

**Affiliations:** 1 Department of Nutrition, Gillings School of Global Public Health, Center for Promotion and Disease Prevention, University of North Carolina at Chapel Hill, Chapel Hill, NC, United States; 2 Granville Vance Public Health, Oxford, NC, United States; 3 Granville Vance Public Health, Henderson, NC, United States; 4 UNC-CH Gillings, Department of Public Health Leadership and Practice, Gillings School of Global Public Health, Chapel Hill, NC, United States; 5 School of Nursing, Duke University, Durham, NC, United States

**Keywords:** academic health department, academic-practice partnership, community health assessment, governmental public health, local health department, organizational innovation, rural health services, rural public health

## Abstract

**Introduction:**

In decentralized public health systems, rural public health departments have fewer economic resources and higher needs for public health programs and services. This case study describes the evolution of a rural district health department’s community and academic partnerships across three phases. Throughout this process, partnerships grounded in community engagement informed the local public health workforce, translated research into practice, and supported the design, implementation, and evaluation of interventions in a rural setting.

**Methods:**

Our approach is guided by three established models—a logic model for evaluating academic health departments (AHD), staged process model of AHD development, and a model of organizational change strategies.

**Results:**

Over a decade of building a rural AHD has resulted in more than $22 million in grant funding, an increase in implemented evidence-based interventions, improved workforce capacity, and expanded formal collaborations with faculty at academic institutions.

**Discussion:**

A mutually shared value of community health improvement and support with a consistent focus on community voice and community health priorities are the threads of success for this rural academic health department model in north-central North Carolina.

## Introduction

### Challenges and opportunities for rural public health

Rural public health has been described as a “complex and under-funded enterprise” which contributes to the well-documented urban–rural divide in the United States, where factors such as educational opportunity, housing quality and availability, and community economic vitality lead to rural residents having poorer health status and worse health behaviors than their urban counterparts (1). Additional rural risk factors driving health disparities include geographic isolation, lower socioeconomic status, limited job opportunities, and limited local access to hospital services, specialists and subspecialists (2). Together these factors contribute to the consistently observed gap between urban and rural premature mortality (1, 3), most commonly from cancer, heart disease, stroke, chronic lower respiratory disease, and unintentional injury (3).

Historically, local health departments (LHDs) in rural settings have been important providers of critical public services including population-based preventive health services, safety-net clinical health care, and maternal and child health (1, 4, 5). In this role, health departments have had “continued responsibility of ensuring reliable and sustainable clinical care services” (1, 4). Rural health departments are smaller, more understaffed, and generally underfunded compared to their non-rural peers, facing additional challenges such as larger geographic areas to cover with fewer staff, difficulty recruiting and retaining staff, lower salaries compared to their non-rural peers, and limited financial resources for basic infrastructure (4).

Given these challenges, rural health departments must rely on innovative strategies to leverage existing resources. One important opportunity to do so is through mutual partnerships with nearby academic institutions. In North Carolina (NC), such opportunities exist with many rural health departments in close proximity to the Gillings School of Global Public Health at the University of North Carolina at Chapel Hill (UNC-CH), the UNC Center for Health Promotion and Disease Prevention (a Centers for Disease Control and Prevention (CDC)-funded Prevention Research Center (PRC)), the Duke University School of Nursing (DUSON), and many others including NC State University and North Carolina Central University. While these opportunities for partnerships exist, there is little current knowledge about how to foster mutually beneficial, long-lasting, and community-focused partnerships across rural health departments and academic institutions. The purpose of this case report is to fill this gap by describing the 12-year development of a Rural Academic Health Department (RAHD) model at Granville Vance Public Health (GVPH), a district health department serving Granville and Vance Counties and the surrounding region in NC.

## Context

### State and local context

North Carolina is one of several southeastern states in which most counties (>70%) are considered rural. North Carolina’s health outcomes roughly parallel national averages (6), though significant portions of the state are located in the CDC’s Diabetes Belt (7) and the National Heart, Lung, and Blood Institute’s (NHLBI) “Stroke Belt” (8).

### District rural health departments in North Carolina

North Carolina’s public health system is considered “decentralized,” placing a large responsibility for funding on LHDs. This is especially true for district (multi-county) configurations, which receive a considerably smaller share of local appropriations compared to single-county departments (9). At the same time, districts have quasi-independent status from county management, reporting directly to a district board of health, which enables greater flexibility in pursuing and managing external funds such as grants (10). Rural counties receive much less revenue from local taxes than urban counties, profoundly affecting the ability to support a wide range of local services including public health. There is an expressed need to leverage financial resources across county lines in rural areas to ensure efficiency and economies of scale. For this reason, during the early 1970s (11), North Carolina encouraged the formation of districts, including GVPH. In 2012, GVPH had a total operating budget of approximately $5,000,000 and nearly 70 personnel. By 2025, GVPH had an approximate $15,000,000 total operating budget and 90 employees.

### Granville Vance public health (GVPH)

GVPH is a rural district health department serving over 100,000 residents across two counties in north central NC, a region with a strong agricultural history and cultural diversity. GVPH primarily serves residents in Granville and Vance Counties, adjacent rural counties located in NC along the Virginia border (12). Both counties are officially designated as “mostly rural” by the US Census, with lower per-capita income, lower educational attainment, and higher rates of uninsured or Medicaid-covered individuals compared to the rest of the state (Table 1) (12–16). The poverty and unemployment rate in Vance County is nearly double the state average, and Vance County is one of just six NC counties where more than half the population identifies as African American (17, 18). GVPH offers mandated public health and prevention programs, full-scale primary care services, Medication for Opioid Use Disorder (MOUD) with integrated behavioral health, environmental health, communicable disease, maternal and child health, family planning, care management, vital records, Women, Infant, and Children (WIC) supplemental nutrition services, immunizations, and health education and promotion programs that respond to local population health needs identified through the Community Health Assessment (CHA) and Community Health Improvement Plan. Carolina Fellows Family Dentistry is a GVPH-affiliated dental clinic in Oxford (Granville County) serving both counties and accepting Medicaid and offering a sliding fee scale. Vance and Granville Counties are both designated as Medically Underserved Areas and Health Professional Shortage Areas for primary care, mental health, and dental services (19).

**Table 1:** Key socioeconomic and demographic factors of a two-county district.

Indicator	Vance county	Granville county	State (NC)
Socioeconomic			
Percent rural	53.3%	71.4%	33.3%
Unemployment rate	4.5%	3.5%	3.6%
Per capita income	$27,223	$33,166	$40,414
Labor force participation rate	58.6%	58.1%	60.3%
Poverty rate	23.2	11.4%	12.8%
Cost-burdened households	31.9%	23.9%	28%
Less than high school	16.9%	14.8%	8.2%
High school graduate (including GED)	34.9%	23.9%	21.7%
Some college, no credential	15.1%	14.9%	11.7%
Bachelor’s degree	14.2%	21.6%	26.4%
Graduate or professional degree	5.7%	8.2%	14.5%
Demographic			
Race/Ethnicity			
Black or African American alone	50.1%	29.8%	21%
Hispanic or Latino	9.8%	11.4%	11.4%
White alone, not Hispanic or Latino	37.4%	55.6%	60.7%
Other race	2.2%	2.1%	5.5%

## Details to understand key programmatic elements of developing a rural academic health department

### Rationale for the innovation of a rural academic health department (RAHD)

AHDs have been formally defined as “an arrangement between an academic institution and a governmental public health agency which provides mutual benefits in teaching, research, and service, with academia informing the practice of public health, and the governmental public health agency informing the academic program” (20, 21). Three core components are evident: (1) a partnership between an academic institution and a governmental health agency; (2) mutual benefits to both public health practice and the academic entity; and (3) sharing of resources such as staff or financial resources (22). AHDs have been described as the public health equivalent of “teaching hospitals” and ideally serve as a site for joint education, research, and practice. The emergence of AHDs followed the 1988 Institute of Medicine (IOM) report “The Future of Public Health” (23), which documented a major disconnect between academic programs in public health and practice settings (24). The motivation for AHD development is similar to that of Prevention Research Centers (PRCs) (25), CDC-funded networks of academic research centers focused on gaining knowledge about the best methodologies for solving the nation’s obstinate health problems (26).

AHDs provide significant value by strengthening evidence-based public health and shortening the 17-year gap between building scientific research and addressing community needs (27). AHDs also promote problem-focused research and serve as incubator sites for public health evidence, while offering LHD employees valuable opportunities for academic development through joint appointments, coursework, and seminars (28). The traditional AHD model has focused on large, non-rural governmental public health (20, 21, 24, 25). Benefits include advancing evidence-based decision-making (28), practice-based research, workforce development, and improved quality of educational experiences (20, 21, 27, 28).

Our RAHD was intended to meet the public health needs of rural residents by connecting local public health practitioners to academic expertise in grant writing and management, program implementation and evaluation, epidemiology, and health disparities research. To accomplish this, the traditional AHD model was adapted using a staged approach to implementation consistent with leading organizational change. The rationale behind this innovative approach originated from a shared value to address the public health needs of rural residents by increasing health department funding and staffing, enhancing workforce development, and elevating community voice through addressing community-identified health priorities. In rural areas, community voices drive priorities, yet funding often stays focused on mandated state services with little remaining for health promotion and wellness. Bringing resources to local rural communities, elevating the practice of local public health, and telling an effective story about the work local community partners accomplish, shape the motivation behind our approach. By aligning funding opportunities with results from our ongoing CHAs, the community remained the center of the RAHD at GVPH, and the reason partners continue to work collaboratively to generate rural public health practice- based evidence.

Several challenges to collaboration across LHDs and academic institutions have been described (21, 29, 30), including perceived differences in mission and agenda, the need for faculty to generate salary support through research grants, the lack of robust approaches to improving evidence-based practice, and the challenge of geographic proximity between rural health departments and academic institutions (24). In building this RAHD, we identified strategies to address each challenge: securing grants to financially support engaged faculty and expanded public health services; starting with an embedded faculty serving as a “Translational and Implementation Research Specialist”; and building local data collection capacity to conduct practice-based rural research.

### Adaptations to build a practice-focused RAHD model

**Figure 1:**
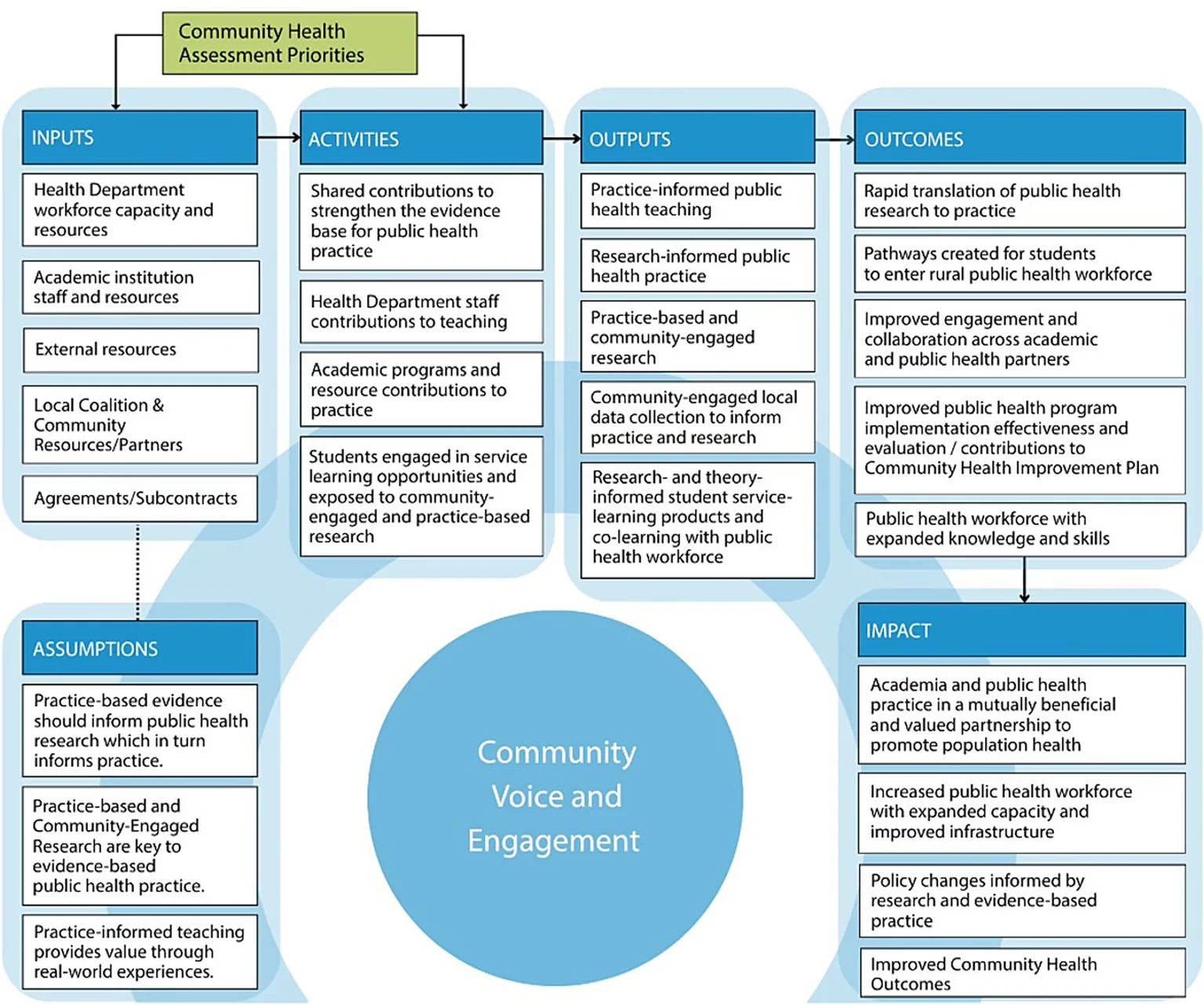
Logic model.

With the focus on public health service to rural communities and drawing from the logic model for evaluating AHDs published by Erwin and colleagues (22, 27), we created an adapted version reflecting our practice- and community-focused components (Figure 1). Adaptations include a focus on *inputs* and *activities* that benefit rural public health practice while leveraging academic and community partnerships. Highlighted are shared contributions to evidence-based public health practice—not just practice-based research—and student learning activities serving as opportunities for community-engaged and practice- based research. *Outputs* were adapted to emphasize the value of practice-informed public health teaching and recognize co-learning between students and public health practitioners. *Outcomes* were expanded to include translation of public health research to practice, with a focus on improving program implementation and effectiveness, enhancing academic-public health partner engagement, and creating pathways for students to enter the rural public health workforce. Common *impacts* of accredited health departments and public health academic programs were replaced with a mutually beneficial academic- public health partnership to promote population health, an increased public health workforce with expanded capacity, and policy changes informed by research and evidence-based practice.

### Staged process of developing a RAHD

**Figure 2:**
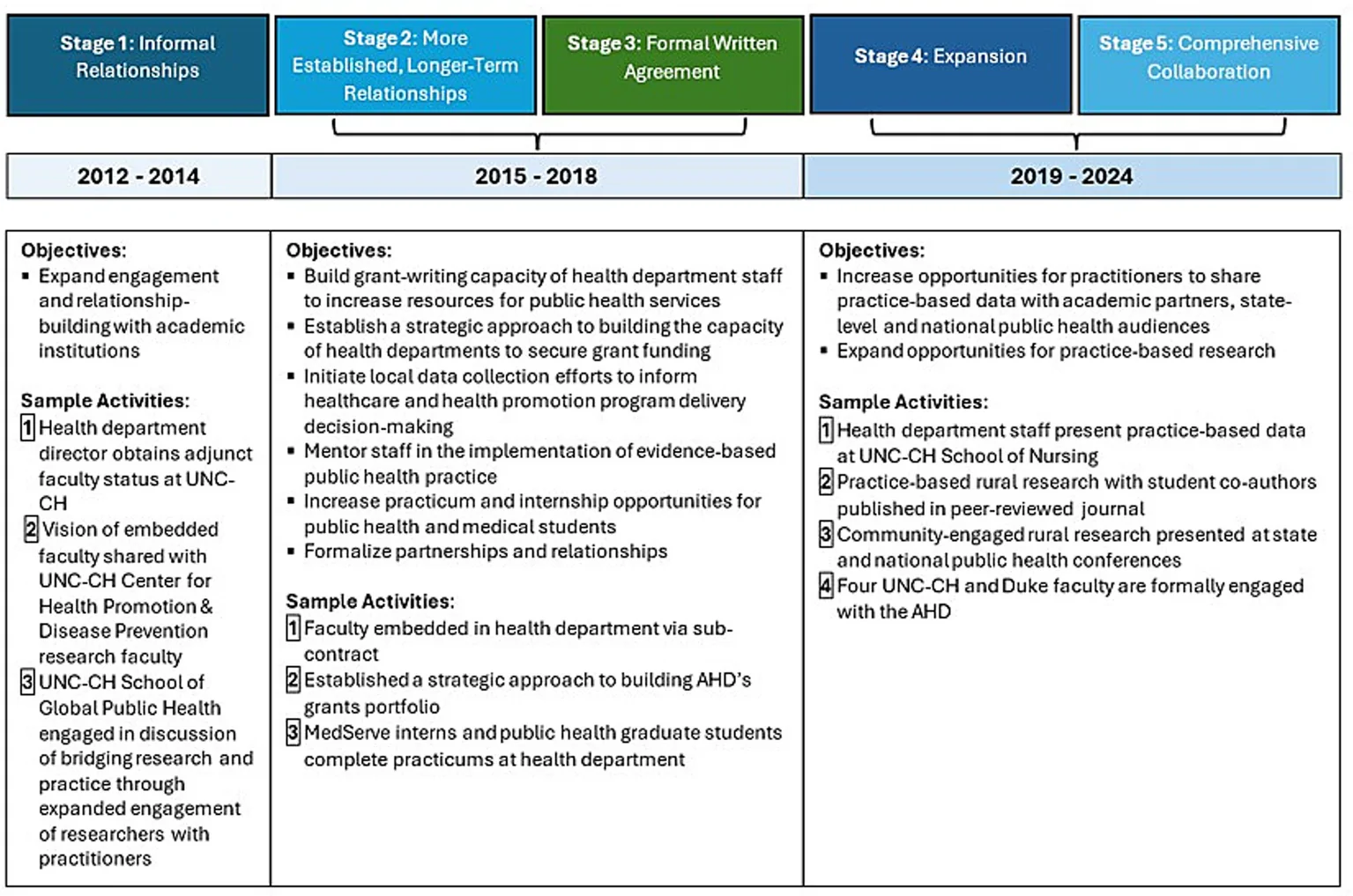
Staged model for implementing a rural academic health department.

A “staged model” has been proposed for AHD development, in which the partnership evolves from informal relationships to formal written agreements and more comprehensive forms of collaboration (31, 32). GVPH’s RAHD is a collaboration that did not emerge overnight; it is a set of partnerships and institutional commitments over a decade in the making. Since 2012, several different relationships and collaborative ventures have gradually built the RAHD into the institution that now exists in 2026. While the RAHD’s development was not linear, its implementation broadly followed the stages of growth detailed in Figure 2. This staged model (32) includes five stages starting with informal relationships, transitioning to formal written agreements, and ending with comprehensive collaboration in education, research, service, shared personnel, and resources. Movement through these five stages took place over 12 years divided into 3- to 5-year periods.

### Stage 1: informal relationships

Informal relationships spanned 2012–2014, beginning the first stage with GVPH partnering with UNC-CH. The GVPH health director began conversations with leadership at UNC-CH Gillings School of Global Public Health and the UNC Center for Health Promotion and Disease Prevention, a CDC-funded PRC. These conversations engaged a UNC faculty member affiliated with both the PRC and School of Public Health who was also seeking opportunities to bring public health research to practice settings. With the health director and faculty member aligned in their vision, the faculty member engaged Gillings leadership to identify an approach to embedding faculty in a LHD, ultimately creating a subcontract to be administered by the School of Public Health’s outreach arm.

### Stages 2-3: more established, longer-term relationships and formal written agreements

In 2015, funds became available with the retirement of a health education supervisor, allowing GVPH to execute the subcontract for the first embedded UNC faculty to serve as a “Translational Research and Implementation Research Specialist,” spending 1 day per week on site. The original vision of the GVPH health director was to create an “embedded research-in-practice role focusing on chronic disease prevention strategies” with an emphasis on grant-writing, workforce development, program evaluation, and consultation on institutional changes. The embedded faculty began by listening to and engaging health department staff to understand GVPH’s organizational context, identify grant-writing assets, and understand staff training needs. While the initial goal was to submit one grant application per year, GVPH applied for seven grants between 2015-2016, and three additional grants from 2016-2017. After the first year, a more strategic approach of prioritizing grant submissions based on health priorities identified in the most recent CHA was implemented. This approach ensured grant funding was responsive to community health priorities and allowed staff hired with grant funding to transition from one grant to another with similar focus.

More established and longer-term relationships were fostered as grant-writing capacity grew. New grant funding created new opportunities for mentoring staff and attracted MedServe interns and UNC-CH students to rural public health practicum and service learning. The increased grant funding also created the need to collect local health data to drive evidence-based health promotion program selection. For example, after GVPH received funding as the regional coordinating site for the evidence-based Diabetes Prevention Program (DPP) (33) focusing on mostly rural racial/ethnic minority populations, qualitative data were collected to adapt program messages and implementation procedures, resulting in achieving full CDC recognition status because local lifestyle training groups met the weight loss performance metric. Similarly, collection of local anthropometric data from nearly 4,000 elementary school children in Granville and Vance counties identified childhood obesity as a public health issue, and a local coalition facilitated by the health department connected community buy-in for evidence-based interventions successfully implemented after local adaptations.

### Stages 4-5: expansion and comprehensive collaboration

As the RAHD continued to grow, it expanded into new activity areas and collaborations with different academic institutions, particularly the DUSON and the Department of Public Health Leadership and Practice (PHLP) at UNC-CH Gillings. While DUSON had a clinical affiliation with GVPH for over a decade, this relationship grew to encompass research and evaluation starting in 2017. DUSON faculty took on a researcher-in-residence role helping with grant writing, research, and telling the story of rural public health. The “DeStress for Health” project, co-led by GVPH and DUSON, involved forming a coalition to address cancer risk locally; multiple community conversations generated a priority on cancer prevention and stress reduction, resulting in new grant proposals. GVPH and DUSON also collaborated on a Health Resources and Services Administration (HRSA) grant seeking to expand pathways for nursing students in rural communities, leading to the hiring of a registered nurse co-located at both institutions. DUSON faculty provided technical support for a HRSA grant developing THRIVe (Treatment, Health services, and Recovery through Integrated care in Vance and Granville counties), a four-year (2023–2027), $4M initiative to enhance youth mental health services. Through the National Clinician Scholar Program (NCSP), GVPH funded postdoctoral scholars for embedded training in public health, with the health director serving on the NCSP advisory board. The Rural Health Equity Hub, funded at Duke to establish learning collaboratives and regional incubators to address rural health equity, identified GVPH as the main community partner.

In fall 2023, GVPH entered a contract agreement with a second UNC-CH Gillings faculty member from the Department of Public Health Leadership and Practice, covering 50% of that faculty’s salary—a contract ongoing in 2026. This was facilitated by the development of a “practice-faculty” position at Gillings that explicitly required faculty to contribute time to practice-related activity. This faculty member served in a grant-funded position designed to improve integration of behavioral health throughout GVPH’s district—into primary care settings, the school system, the criminal justice system, and across the faith-based community. Both UNC-CH Gillings faculty members served on GVPH’s CHA internal steering committee. GVPH’s work has been highlighted in several UNC-CH Gillings courses, students have completed internship opportunities, and presentations have elevated GVPH’s RAHD work. For the past 2 years, outcomes of this collaboration have been showcased at the American Public Health Association conference. GVPH staff and faculty partners also regularly prepare abstracts for the National Association of County and City Health Officials (NACCHO) and the NC Public Health Association.

### Strategies used to lead organizational change

The initial years of leading change to build this RAHD were more art than science. While we did not explicitly employ Kotter’s model (34) at the time, in retrospect we leveraged several of its elements: (1) creating a strategic vision, (2) enabling action by removing barriers, (3) generating quick wins, (4) sustaining early gains, and (5) institutionalizing changes. With both the health director and embedded faculty having years of public health experience, they leveraged experiential and empirical knowledge to *form a strategic vision* to bring more resources to the health department through grant writing and technical assistance. Strategically targeting grant funding aligned with CHA priorities allowed multiple funding sources to support health department staff while remaining responsive to community health priorities.

*Enabling actions by removing barriers* involved organizing grant preparation tasks and identifying skills and training needs for staff who would take on grant-writing responsibilities over time. The faculty member’s consultation with health department staff to capture practice-specific information removed barriers and created a more practice-based research approach. University resources—including access to researchers, journal articles, and health research databases—were central to the grant-writing process. In the first year of having an embedded faculty member, GVPH secured over $1 million from seven new grants, a *quick win* that far exceeded the goal of submitting one grant in the first year. The grant-writing process developed in 2014 is still used by health department staff today.

*Early gains were then consolidated* with workforce training and capacity development, providing mentoring for MedServe interns, enabling practitioners to present their work at universities, collaborating with academic partners on staff and community training, pairing public health students with practitioners for co-learning through practicums, and sharing practice-based research findings through peer-reviewed publications. Now, more than a decade later, these changes are fully integrated into GVPH’s systems, processes, and work environment.

## Results

**Figure 3:**
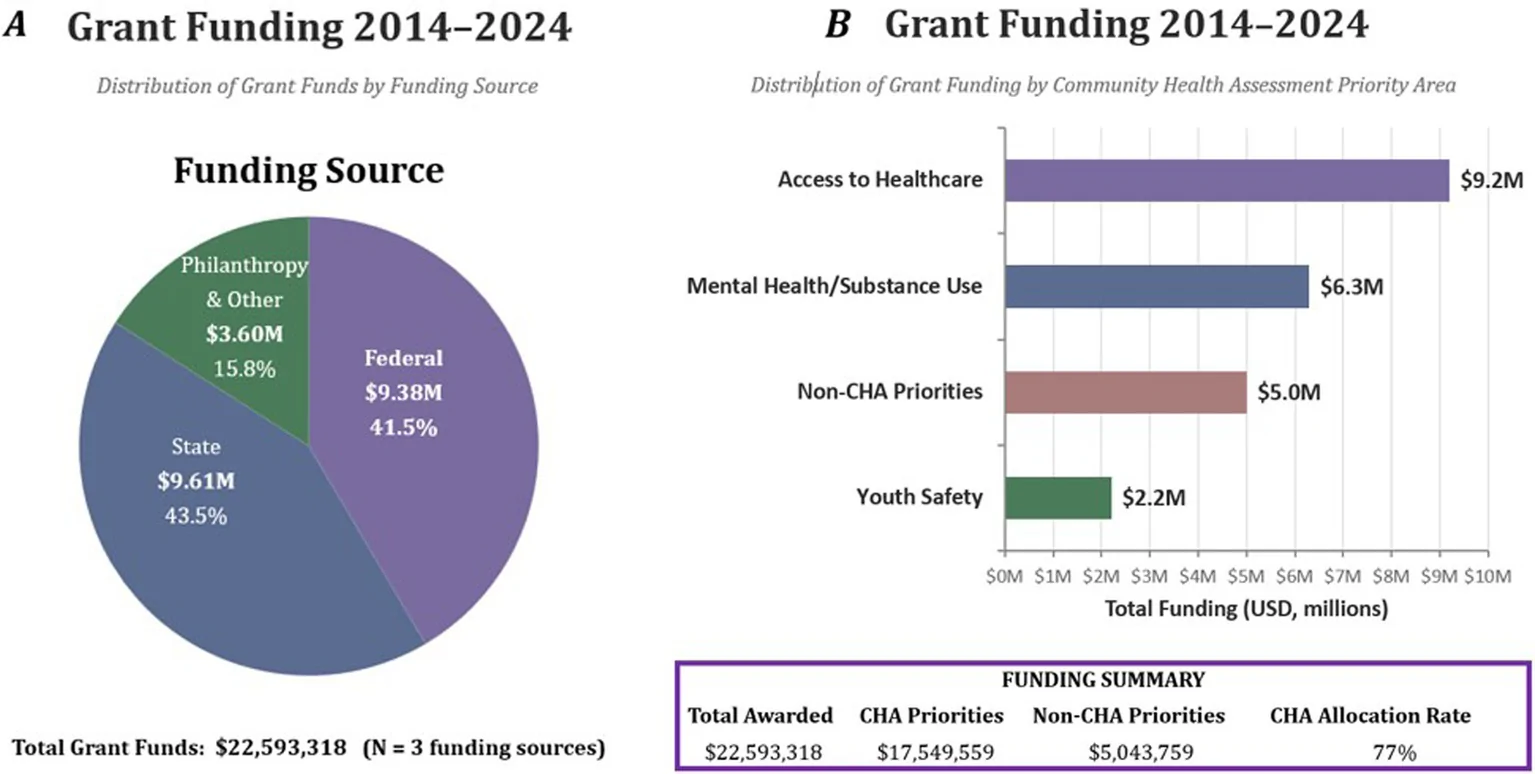
Grant funding 2014–2024. Federal sources include but are not limited to Health Resources and Services Administration (HRSA), Office of Health Equity (OHE), and similar agencies. State sources include but are not limited to North Carolina Department of Health & Human Services (NC DHHS), North Carolina Office of Rural Health (NC ORH), and North Carolina General Assembly appropriations. Philanthropy/other sources include Triangle North Healthcare Foundation (TNHF), Kate B Reynolds Charitable Trust (KBR), Duke Endowment, Duke University.

The *outputs* of this RAHD model are evident in numerous grants secured (Figure 3), publications and presentations documenting GVPH’s innovative community work, internship experiences for undergraduate and graduate students, and new and ongoing projects formed through partnership with the local community, the health department, and affiliated universities (35). In Figure 3, the distribution of grants funded between 2014–2024 by CHA priority areas and funding sources is depicted. Over this 10- year period, GVPH was awarded over $22 million in grants, with 77% funding CHA priority areas. Funding sources included federal (41.5%), state (42.6%), and philanthropic or other sources (15.9%). These practice-academic partnerships produced more peer-reviewed products than in earlier years (pre- 2014), including national and state conference abstracts as well as journal articles with community members, health department, and university student co-authors (36). Grant funding created more opportunities for public health students and clinical interns and expanded GVPH’s capacity to implement evidence-based interventions over the decade (e.g., DPP (33), Centering Pregnancy (37), CATCH (Coordinated Approach To Child Health) (38), NAP SACC (Nutrition and Physical Activity Self-Assessment for Child Care) (39), reaching a larger proportion of rural residents. Other outputs included expanded formal collaborations with academic partners—from one embedded faculty position in 2015 to four in 2026—and increased staff from 70 in 2012 to 90 in 2025. Overall, this RAHD partnership led to grant-funded programs, services, and staff that contributed to the tripling of the health department’s overall budget and increased capacity to improve public health practice in the rural communities GVPH serves.

These outputs resulted in *outcomes* that include improved workforce capacity in grant-writing, delivery and implementation of clinical and health promotion/disease prevention interventions, enhanced program evaluation skills, and increased skills in community-partnered data collection using qualitative and quantitative methods. The *impact* of over 10 years of building a RAHD is captured in achieving the goal of increasing the revenue of a rural health department to better serve its population, improved evidence-based public health practice, and expanded formal collaboration between a rural health department and academic partners to address local public health problems. Furthermore, during the period in which the RAHD expanded its programs and services in partnership with community organizations and state-level initiatives, county-level health indicators showed improved health care access, lower infant mortality and teen pregnancy rates, and decreased mortality from suicide, drug poisoning, and drug overdoses (40, 41).

## Discussion: expanding and replicating the model

Academic Health Departments have the potential to strengthen ties across academics and practitioners, speed the translation of research to practice, build capacity, and create a sustainable pathway for students to enter the public health workforce. Over the last 12 years, our RAHD has made faculty connections across several nearby universities, secured additional grant funding to pursue innovative public health programming, and documented the success of this programming through numerous publications and presentations. The GVPH experience may have policy and practice implications for other rural health departments interested in replicating this model. Replication provides mutual benefit to the health department and academic institution, but it takes sustained effort on both sides to maintain the relationships that ultimately uphold the model.

Lessons learned from this experience may inform future replication efforts and identify policy levers that support implementation of the model. First, investments of time, talent, and funding are key to sustaining a RAHD. Having people who prioritize and understand the importance of the academic-health department relationship for advancing research and practice is critical, and these investments can catalyze and support efforts until sustainable funding streams are built. Second, RAHD models elevate public health practice through bidirectional learning activities, which should be the mainstay of all academic health department work, especially in rural areas needing a clear pathway from student to rural health department roles. Bidirectional workforce development is critical to the academic health department model, increasing professional development opportunities for staff and creating pathways for student exposure to health department roles. Given documented provider shortages and limited access to development for rural staff (42), learning activities should include exposure of students to public health leadership roles in rural settings. Health department employees should also benefit from professional development opportunities and academic appointments affording access to university learning resources. From a research perspective, rural AHDs offer a rich environment to develop evidence translatable to practice, and both academic research grants and public health funding opportunities—pursued with rural public health relevance in mind (21)—should be encouraged to further align institutional resources.

Our experience also brings forward multiple recommendations for replicating this model. Incentivizing academic partnerships through seed funding to support local relationship building and securing subsequent grant funding and training grants can help sustain and solidify infrastructure that supports collaboration and AHD model goals. These funding opportunities should be earmarked for explicit replication, allowing support over time, sustainability planning, and outcome evaluation. Academic institutions have a significant role to play in advocating for RAHD funding. Current efforts include development of a Practice Strategic Plan (43) and creation of an Associate Dean of Practice position at UNC Gillings and a Director of Rural Health at Duke School of Nursing. Given clear reciprocal benefits for academic entities and affiliated health systems, institutions should actively engage in policy advocacy to strengthen the financial foundation for public health infrastructure and workforce development through RAHDs. Moreover, partnerships can be broadened beyond schools of public health and nursing to include policy, data science, climatology, social work, and engineering—disciplines critical to training the next generation of practitioners to address contemporary and emerging public health challenges within the nuance of local political and interpersonal contexts. LHD leadership can advocate for funding and amplify the impact of the RAHD through university service and leadership roles, including participation on Practice Advisory Councils and Advisory Boards.

From a policy perspective, incentivizing academic-practice partnerships is critical, and these partnerships should be formally tied to accreditation processes for both academic institutions and LHDs to embed their value structurally. Creating standardized toolkits and playbooks, including template Memoranda of Agreement (MOAs), would ease the process of establishing new RAHDs (44). Academic and public health partnerships must also be explicitly recognized in promotion and tenure processes, signaling institutional value. Community-informed research remains central to addressing real-world public health challenges. Building the next generation of public health researchers who can work collaboratively with practitioners requires a multifaceted approach. Practice-based Research Networks (PBRNs) offer a pragmatic infrastructure supporting collaboration between public health and academia (45). PBRNs are networks “united on the goal of expanding the science of clinical care through systematic investigation” that could be replicated for public health practice, offering a pragmatic approach to collectively build infrastructure and maintain the research side of the academic health department model. Regional networks may be especially valuable in under-resourced rural counties given workforce challenges. Funding specifically for public health PBRNs can support infrastructure development, sustainability, and innovation, particularly for community-engaged research, which often requires substantial time and remains under-resourced in rural areas (1, 46).

In conclusion, the RAHD offers a valuable model for bridging academia and public health practice in rural settings where resources are lacking and workforce development and innovation are urgently needed. This community-focused experience demonstrates the value of sustained investment, bidirectional learning, and strategic partnerships in advancing public health goals. Replication of this model requires intentional funding, investment of time and talent, trust with community partners, and institutional commitment from both academia and public health. Leveraging broader multi-sectoral collaborations and existing networks will be key to long-term sustainability.

## Data Availability

The original contributions presented in the study are included in the article/supplementary material, further inquiries can be directed to the corresponding author.
